# Comparative Analyses of Mitochondrial Genomes Provide Evolutionary Insights Into Nematode-Trapping Fungi

**DOI:** 10.3389/fmicb.2020.00617

**Published:** 2020-04-15

**Authors:** Ying Zhang, Guangzhu Yang, Meiling Fang, Chu Deng, Ke-Qin Zhang, Zefen Yu, Jianping Xu

**Affiliations:** ^1^State Key Laboratory for Conservation and Utilization of Bio-Resources in Yunnan, and Key Laboratory for Southwest Microbial Diversity of the Ministry of Education, Yunnan University, Kunming, China; ^2^School of Life Sciences, Yunnan University, Kunming, China; ^3^Department of Biology, McMaster University, Hamilton, ON, Canada

**Keywords:** entomopathogenic fungi, anamorphic Orbiliaceae, mitochondrial phylogeny, adaptive evolution, rps3

## Abstract

Predatory fungi in Orbiliaceae (Ascomycota) have evolved a diversity of trapping devices that enable them to trap and kill nematodes, other small animals, and protozoans. These trapping devices include adhesive hyphae, adhesive knobs, adhesive networks, constricting rings, and non-constricting rings. Their diversity and practical importance have attracted significant attention from biologists, making them excellent model organisms for studying adaptative evolution and as biological control agents against parasitic nematodes. The putative origins and evolutionary relationships among these carnivorous fungi have been investigated using nuclear protein-encoding genes, but their patterns of mitogenome relationships and divergences remain unknown. Here we analyze and compare the mitogenomes of 12 fungal strains belonging to eight species, including six species representing all four types of nematode trapping devices and two from related but non-predatory fungi. All 12 analyzed mitogenomes were of circular DNA molecules, with lengths ranging from 146,101 bp to 280,699 bp. Gene synteny analysis revealed several gene rearrangements and intron transfers among the mitogenomes. In addition, the number of protein coding genes (PCGs), GC content, AT skew, and GC skew varied among these mitogenomes. The increased number and total size of introns were the main contributors to the length differences among the mitogenomes. Phylogenetic analyses of the protein-coding genes indicated that mitochondrial and nuclear genomes evolved at different rates, and signals of positive selection were found in several genes involved in energy metabolism. Our study provides novel insights into the evolution of nematode-trapping fungi and shall facilitate further investigations of this ecologically and agriculturally important group of fungi.

## Introduction

Nematode-trapping fungi (NTF) are a taxonomically heterogeneous group of asexual ascomycetes that can form special structures (traps) to capture free-living nematodes in soil (Barron, 1977). Members of the Orbiliaceae family represent the largest group of NTF, which include at least 96 species belonging to genera *Arthrobotrys* (53 species, producing adhesive three-dimensional networks), *Dactylellina* (28 species, producing adhesive knobs, non-constricting rings, and adhesive column), and *Drechslerella* (14 species, producing constricting rings) (Zhang and Hyde, 2014). These NTF species were originally defined based primarily on conidial characteristics such as size, septation, and type of conidiogenous cells (Subramanian, 1963). Recent research showed that trapping devices are also phylogenetically informative for classifying NTF species (Rubner, 1996; Scholler et al., 1999; Li et al., 2005; Yang and Liu, 2006; Yang et al., 2007).

Because of their unique life style and potential as biocontrol agents against parasitic nematodes, NTF have been studied over several decades. Those studies cover a range of topics, including their substrate preference, the origin and development of trapping devices, and the molecular mechanisms of pathogenesis against nematodes. Previous studies based on multiple nuclear gene phylogeny indicated that the trapping mechanisms within the Orbiliales have evolved along two major lineages, one leading to species with constricting rings and the other to species with adhesive traps, including three-dimensional networks, adhesive knobs, and adhesive branches (Yang et al., 2007). Furthermore, molecular clock calibration based on two fossil records estimated that the two major lineages diverged from each other ∼246 million years ago (Mya) (Yang et al., 2012).

So far, the genome sequences of several NTF have been obtained, assembled, and annotated, including strains from *Arthrobotrys conoides, Arthrobotrys oligospora*, *Dactylellina appendiculata, Dactylellina drechsleri, Dactylellina haptotyla, Drechslerella stenobrocha*, and *Dactylellina cionopaga.* Such a rich dataset has made it possible for evolutionary studies at the genomic level (Zhang W. et al., 2016). Genome sequence comparisons have shown that NTF tend to have expanded gene families coding chitin-degrading enzymes and proteases, possess a well-developed cellulose-degrading metabolism, but have relatively few plant pathogenesis-related genes. Together, these features are consistent with their saprotrophic ancestry (Liu et al., 2014). However, nothing is known about the evolutionary patterns and divergences of NTF from the mitochondrial genomic perspective.

Mitochondria are energy-generating organelles that play a critical role in numerous cellular functions, including ATP production, cellular homeostasis, and apoptosis (Wallace, 2005). Due to their distinct genome structure, inheritance pattern, and rates of evolution, mitochondrial genomes (mitogenomes) have been frequently used in evolutionary biology and systematic studies (Pantou et al., 2006; Kouvelis et al., 2008; Basse, 2010). However, among the estimated 2.2–3.8 million fungal species (Hawksworth and Lücking, 2017), only 375 records of fungal mitogenomes are published by the end of 2019^1^. These mitogenomes show a large variation in genome size, ranging from 12,055 bp in *Rozella allomycis* to 19,431 bp in the fission yeast *Schizosaccharomyces pombe* (Chen and Butow, 2005) and 235,849 bp in the common filamentous plant pathogen *Rhizoctonia solani* (Losada et al., 2014). Despite the large genome size variation, the fungal mitogenomes usually contain 14 genes that encode oxidative phosphorylation system proteins, the large (rnl) and small (rns) ribosomal RNA subunits, and a fairly constant set of tRNA genes (Gray et al., 1999; Lavín et al., 2008). The major contributor to the large genome size variations among the fungal mitogenomes are the varying number of introns, as well as the genes within those introns such as the endonuclease genes containing either the GIY-YIG or the LAGLIDADG motifs (Wu et al., 2009; Mardanov et al., 2014; Sandor et al., 2018). So far, most of these mitogenomes have been reported separately and there have been very few comparative analyses of mitogenomes of a specific group of fungi, including NTF.

In this study, we analyzed the complete mitochondrial genomes of six NTF species representing all four types of nematode trapping devices and two other closely related species incapable of forming any nematode trapping devices. To provide a better understanding of the mitogenomic diversity among NTF, for two species *A. oligospora* and *Drechslerella brochopaga*, we also analyzed the mitogenomes from four and two natural isolates, respectively. The 12 mitogenomes were annotated, and their gene contents, structures, and gene orders were compared to assess variation and conservation among NTF strains and species. All the mitogenomes we analyzed are largely syntenic, but the size and the intron content differ greatly. The Ka/Ks ratios obtained clearly suggested that though purifying selection was the dominant force driving the evolution of most protein-coding genes, signals of positive selection were found for several genes across NTF species. Our comparative analyses of mitogenomes of NTF provide an important foundation for future studies on NTF population genetics, taxonomy, mechanisms of trap formation, and biocontrol application.

## Materials and Methods

### Sampling, DNA Extraction, and Genome Sequencing

The published mitochondrial genomes of six NTF species representing all four types of nematode trapping devices were analyzed and compared with each other as well as with two closely related species but are incapable of producing any nematode trapping devices (Jiang et al., 2018; Zhou et al., 2018; Deng and Yu, 2019a, b; Fang et al., 2019; Li and Yu, 2019; Wang S. et al., 2019; Zhang and Yu, 2019). In addition to the nine published mitogenomes, we generated the mitogenome sequences of three more natural strains of *A. oligospora* and included them for both inter- and intra-species comparisons among NTFs (Table 1). All the strains and species were identified based on their morphological features and their sequences at the internal transcribed spacer (ITS) regions of the ribosomal RNA gene cluster (Zhang Y. et al., 2016). To obtain their mitogenome sequences, their total genomic DNA was extracted from the mycelia collected from single-spore cultures growing on cellophane membrane on PDA according to the method described by Zhang et al. (2011). Sequencing libraries and the whole genome sequencing were performed with an Illumina HiSeq 2500 Platform by Novegene Co., Ltd. (Beijing, China).

**TABLE 1:** Mitogenome features in six nematode-trapping fungal species and two related non-predatory species.

Species	Trapping devices*	Strains	Gen Bank No.	Genome Size	GC content(%)	Intron size	Exon size	Intron No.																Tot.	tRNA No.	ORF	Intron I	Intron II
								cob	cox1	cox2	cox3	nad1	nad2	nad3	nad4	nad4L	nad5	nad6	atp6	atp8	atp9	rps3	rnpB					
*Arthrobotrys oligospora*(AO83)	AN	YMF1.01883	MK571436	160613	24.97	71121	12249	3	12	7	3	2	4	0	2	0	1	0	1	0	0	1	0	35	24	41	20	0
*Arthrobotrys oligospora*(AO75)	AN	YMF1.02775	MN977365	155181	25.15	78430	12982	3	12	7	3	2	3	0	2	0	2	0	2	0	0	0	0	36	26	49	8	0
*Arthrobotrys oligospora*(AO65)	AN	YMF1.02765	MN977364	159392	25.14	80982	13157	4	15	7	3	2	2	1	2	0	3	0	2	0	0	0	0	41	25	63	14	0
*Arthrobotrys oligospora*(AO37)	AN	YMF1.03037	MN977366	165815	24.86	79479	12640	2	11	6	3	2	3	0	2	0	2	0	1	0	0	0	0	32	25	53	12	0
*Arthrobotrys musiformis*(AM)	AN	YMF1.03721	MK547645	179060	24.61	82220	14055	4	13	1	5	3	0	0	2	0	7	1	2	0	1	0	0	39	18	51	23	2
*Drechslerella brochopaga*(DB29)	CR	YMF1.01829	MK820635	280699	26	177801	12948	4	12	4	9	3	4	0	2	0	9	1	2	0	1	1	1	51	28	77	9	1
*Drechslerella brochopaga*(DB16)	CR	YMF1.03216	MK550698	193195	25.84	91902	12053	5	7	5	4	0	1	1	0	0	5	0	0	0	0	1	0	28	22	59	16	0
*Dactylellina leptospora*(DL)	AK&NCR	YMF1.00042	MK307795	201677	25.11	101927	12594	5	7	8	8	4	6	1	2	0	5	0	3	0	1	0	0	50	25	71	21	1
*Dactylellina haptotyla*(DH)	AK&NCR	CBS200.50	MK554671	146101	22.92	63920	12834	5	7	4	4	1	1	1	1	0	5	1	2	0	0	0	1	32	25	42	23	0
*Dactylellina cionopagum*(DC)	AC	YMF1.00569	MK307796	194125	26.6	96838	13675	7	11	2	7	4	4	1	2	0	4	0	3	0	1	0	1	46	25	56	25	2
*Dactylella tenuis*(DT)	NONE	YMF1.00469	MK820634	186056	26.21	89297	12660	6	8	3	8	0	5	1	1	0	7	0	1	0	1	1	1	41	24	51	20	1
*Dactylella dorsalia*(DD)	NONE	YMF1.01835	MK547647	191042	25.3	107237	12361	6	17	6	6	2	5	0	2	0	8	1	2	0	0	0	1	55	25	85	26	0

*AN, adhesive networks; AK & NCR, adhesive knobs and non-constricting rings; CR, constricting rings; AC, adhesive column; NONE, no trapping device produced.

### Assembly and Annotation of Mitochondrial Genomes

The sequencing, assembly, and annotation for nine of the 12 mitogenomes analyzed in this study have been described previously (Jiang et al., 2018; Zhou et al., 2018; Deng and Yu, 2019a, b; Fang et al., 2019; Li and Yu, 2019; Wang S. et al., 2019; Zhang and Yu, 2019). To assemble and annotate the three remaining mitogenomes of *A. oligospora* strains, their mitogenome sequence reads were first identified for homology with the published *A. oligospora* mitogenome (GenBank accession number MK571436) using the NCBI BLAST algorithm. For each strain, all mitogenome reads were extracted from the whole genome sequencing data according to our previously described methods (Deng and Yu, 2019a). We then used the SPAdes 3.9.0 software with a kmer size of 17 to *de novo* assemble the three *A. oligospora* strains’ mitogenomes with the obtained clean reads (Bankevich et al., 2012). The gaps were filled by separate PCR and sequencing with primers on regions flanking the gaps, resulting in one circular mitochondrial genome for each strain. The mitogenomes were annotated using both MFannot^2^ and GLIMMER^3^. The tRNAs were identified and annotated using tRNAscan-SE (Brooks and Lowe, 2005). All ORFs were searched and identified by ORFFinder^4^. Syntenic blocks in all 12 mitogenomes were identified using MAUVE 2.3.1 based on whole mitogenome sequence alignments (Darling et al., 2004). For each strain, we determined the relative synonymous codon usage (RSCU) in 14 mitochondrial protein coding genes (PCGs) using CodonW1.4.2^5^ with the fungal mitochondrial genetic code 4. The following formulas were used to assess mitogenome strand asymmetry: AT skew = [A − T]/[A + T]; GC skew = [G − C]/[G + C].

The non-synonymous (Ka) and synonymous substitution rates (Ks) for all 14 mitochondrial PCGs of the NTF mitogenomes were calculated using KaKs_Calculator2.0 (Wang et al., 2010). The calculated Ka/Ks ratios were then analyzed to infer the potential selection pressure on each gene pair. Generally, a Ka/Ks ratio greater than, equal to, or less than 1 indicates positive (diversifying) selection, neutral evolution, or purifying (negative) selection, respectively.

Transition transversion biases were estimated for both the mitogenomic PCGs and the three single-copy nuclear protein-coding genes: the second largest subunit of RNA polymerase II gene (*rpb2*), the translation elongation factor 1-α gene (*tef1-*α), and the β tubulin gene (β*-tub*). These estimates were obtained by employing Maximum Composite Likelihood (MCL) statistical method with the Tamura-Nei model and gaps/missing data were excluded. In addition, we also obtained the Maximum Likelihood estimation of transition transversion biases by employing Kimura 2-parameter substitution model, where evolution rates were set as Gamma distributed with Invariant sites (G + I) and the number of discrete gamma categories was 5. The transition transversion bias estimates by MCL and ML were carried out in MEGA6 (Tamura and Nei, 1993; Tamura et al., 2013).

### Phylogenetic Analysis

To determine the evolutionary relationships of our selected NTF and other species from the phylum Ascomycota, amino acid sequences at each of the 14 PCGs (*atp6, 8–9, cob, cox1–3, nad1–6*, and *nad4L*) were individually analyzed for all selected taxa. The 14 gene trees were compared among themselves for their tree topology congruences. As there was no evidence of phylogenetic incongruence, the concatenated amino acid sequences of these 14 PCGs were used to construct a more robust phylogeny among the taxa. Here we included 94 species from other representative phyla and classes, including Pezizomycotina, Saccharomycotina, Taphrinomycotina, Zygomycota, Blastocladiomycota, and Basidiomycota. Our 14 individual gene trees showed no evidence of significant incongruence. Thus, the multiple protein sequences were concatenated and aligned using MUSCLE in software MEGA6.0 (Edgar, 2004; Tamura et al., 2013). MrBayes v3.2.6 (Huelsenbeck and Ronquist, 2001) was used to construct the phylogenetic tree using the Bayesian inference (BI) method based on the combined gene set. In this phylogenetic analysis, the first 25% trees were discarded as burn-in, and the remaining trees were used to calculate Bayesian posterior probabilities (BPP) in a 50% majority-rule consensus tree.

## Results

### Genome Size and Intron Distribution

The mitogenomes of the 12 fungal strains investigated here were all composed of circular DNA molecules with genome sizes ranging from 146,101 bp to 280,699 bp (Table 1). The two species incapable of forming trapping devices have mitogenomes sized 186,056 bp and 191,042 bp, respectively, well within the range of the mitogenomic sizes of those with nematode trapping ability. However, despite their large mitogenome size difference (almost two-fold difference between the largest and the smallest mitogenomes), the concatenated lengths of all exons were very similar among the strains, from DB16’s 12,053 bp to AM’s 14,055 bp, a difference of about 2 kb. Indeed, strain DB29 has the largest mitogenome at 280,699 bp but an intermediate concatenated exon size of 12,948 bp. We note that the concatenated exons excluded those in the open reading frames (ORFs) of introns. Indeed, some of the introns contain ORFs coding for potential endonucleases (group I) or reverse-transcriptases (group II). Most of the mitochondrial introns we annotated in these 12 strains belong to group I, only 1 or 2 introns distributed in strains AM21, DB29, DL42, DT, and DC are group II introns.

**FIGURE 1:**
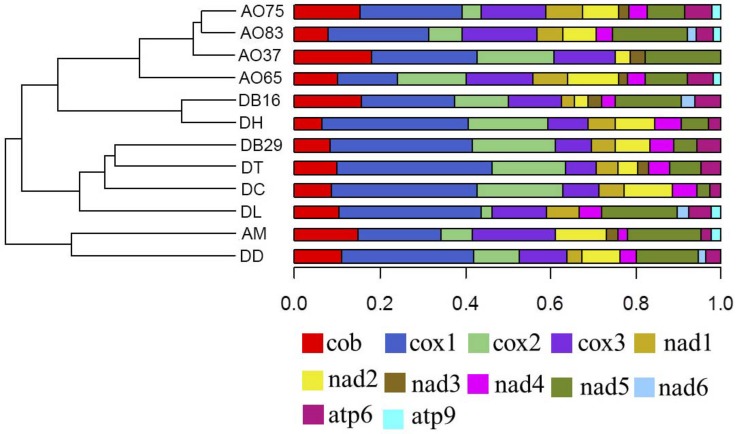
Dendrograms showing the relationships among strains based on their relative intron numbers in each of the 14 mitochondrial core protein coding genes. The “plotTree.barplot” function from the *phytools* R package (Revell, 2011) was used to estimate the similarities between samples. A distance matrix was first calculated using the intron numbers distributed in the 14 protein coding genes within each strain.

Among the 14 protein coding genes (*cox1, cox2, cox3*, *cob*, *atp6, atp8, atp9*, *nad1, nad2, nad3, nad4, nad4L, nad5, nad6*), 12 contained at least one intron in one of the 12 strains (Table 1). The gene containing the most introns was *cox1*. However, two genes, *nad4L* and *atp8*, have no intron in any of the 12 strains. The variations in intron numbers were found both among species as well as among strains within species, in this case *A. oligospora* and *Dr. brochopaga*. Overall, though there are differences in how introns are distributed among species, there is no clear pattern for association between the total number of introns and specific type of trapping devices (Figure 1). Our results are consistent with the dynamic nature of intron distribution in fungal mitogenomes.

### Gene Arrangement Analysis

**FIGURE 2:**
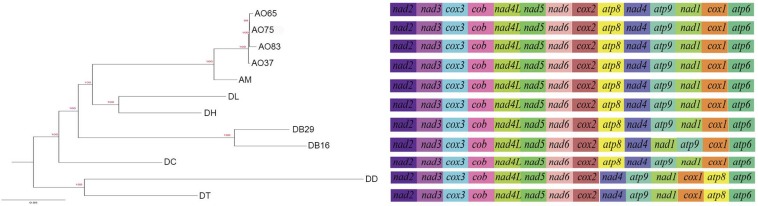
Phylogeny of nematode trapping fungi and their close relatives based on their concatenated nucleotide sequences of the 14 mitochondrial core protein coding genes. The gene order in each mitogenome is indicated.

**FIGURE 3:**
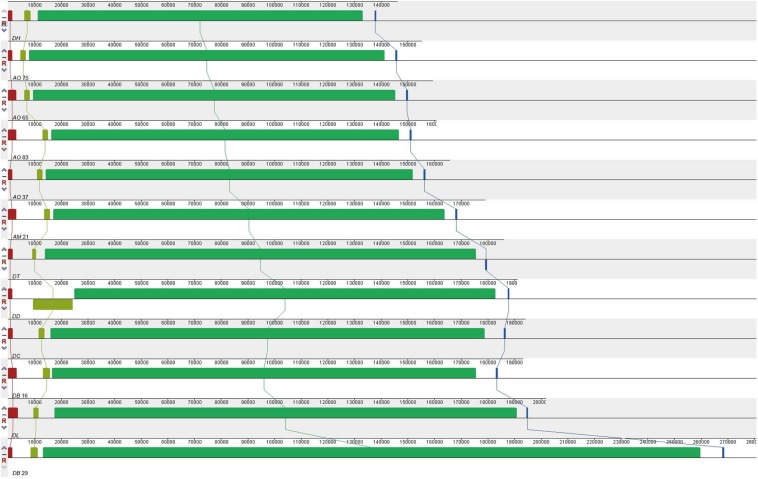
Co-linearity analysis of 12 fungal mitogenomes analyzed in this study, generated with Mauve 2.3.1.

All the mitogenomes from the eight fungal species encode an essential set of conserved genes including three cytochrome c oxidase subunits (*cox1, cox2, cox3*), apocytochrome b (*cob*), three ATP synthase subunits (*atp6, atp8, atp9*), seven subunits of NADH dehydrogenase (*nad1, nad2, nad3, nad4, nad4L, nad5, nad6*), the small and large ribosomal RNA subunits (*rns, rnl*). The mitogenome-encoded ribosomal small subunit protein 3 (*rps3*) and the ORF coding for a putative *rnpB* were also found among our strains. The number of tRNA genes in the mitochondrial genomes of the NTF species ranges from 18 to 28. All the genes are located on the same DNA strand. The gene order of the 14 major PCGs was highly conserved across the mitogenomes of the six NTF species, with the exception of switched locations of *atp9* and *nad1* genes in DB16 (Figure 2). However, compared to the six NTF species, the two non-predatory fungal species DD and DT had a large rearranged mitogenome region, with *atp8* being from the upstream of the *nad4* gene changed to the downstream of *cox1* gene. Based on the gene orders, the 12 mitogenomes could be divided into three types as shown in Figure 2. The first type contains the four strains of *A. oligospora* and the representative of each of the following species AM, DL, DH, DC, as well as DB29. The second type is represented by DB16, and the third type by DD and DT. The high gene synteny among these mitogenomes is also reflected by the Mauve alignment (Figure 3). All 12 mitogenomes could be divided into four homologous regions. The red, light green and blue regions are all similar in their sizes, except for the large light green region in DD that is also inverted compared to all other strains. In contrast, the sizes of the largest syntenic regions (in dark green) differed substantially among these species according to their mitochondrial genome sizes (Figure 3).

### AT and GC Skews

**FIGURE 4:**
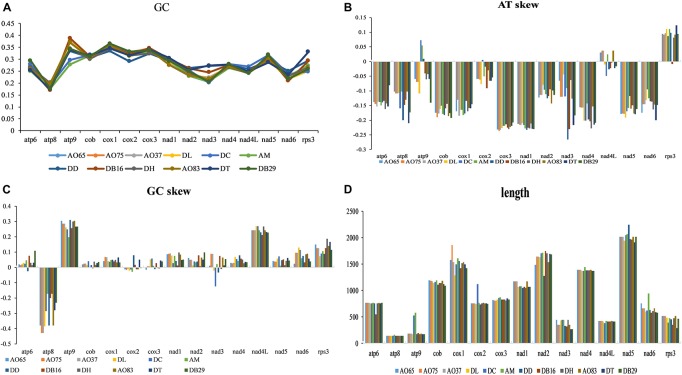
Variation in the length and base composition of each of the 15 protein-coding genes among 12 fungal mitochondrial genomes. **(A)** GC content across the 15 genes; **(B)** AT skew; **(C)** GC skew; **(D)** gene length variation.

The lengths and GC contents of the 15 core PCGs varied among the 12 mitogenomes (Figure 4). The low GC content of all 12 strains (ranging from 22.92% to 26.6%) is similar to other fungal mitogenomes in Pezizomycotina (Lin et al., 2015). Here, the mitogenome from DC contained the highest GC content (Supplementary Table S1). Our analyses identified that the AT skews of most genes in most of our samples were negative. The only exceptions were the *rps3* gene in most strains (Supplementary Table S2), the *atp9* gene in three samples (DC, AM, and DD), and the *nad4L* gene in five samples (all AO samples and AM). Conversely, GC skew was positive in most PCGs except at *atp8* gene in all strains, and the *cob, cox2-3*, and *nad3* genes for some of the strains. Our results suggested that the nucleotide compositions varied among NTF species and PCGs. Interestingly, in contrast to the skews calculated for other species of NTF, all adhesive network- producing strains have more Ts than As in their protein coding genes, indicating that their mitogenomes were likely subjected to different selection pressures than other NTF species and the two non-predatory species.

### Codon Usage Analysis

**FIGURE 5:**
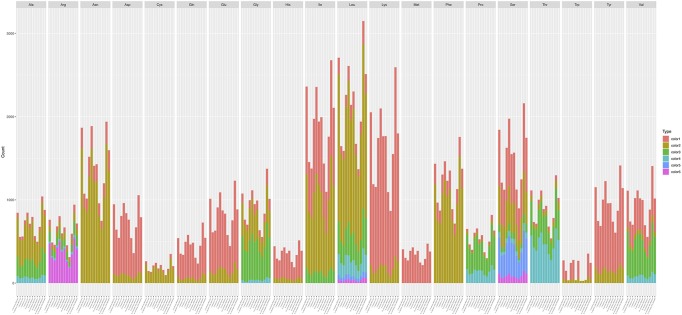
Codon usage in the 12 fungal mitochondrial genomes. Frequency of codon usage is plotted on the *y*-axis.

Codon usage analysis indicated that the most frequently used codons were UUU (for Phenylalanine; Phe), UUA (for Leucine; Leu), AUU (for Isoleucine; Ile), AUG (for Methionine; Met), GUU (for Valine; Val), and AGA (for Serine; Ser), CCU (for Proline; Pro), ACU (for Threonine; Thr), GCU (for Alanine; Ala), UAU (for Tyrosine; Tyr), CAU (for Histidine; His), GGU (for Arginine; Arg), AAU (for Asparagine; Asp), UGU (for Cysteine; Cys), CAA (for Glutamine; Gln), GAA (for Glutamic acid; Glu), GGA (for Glycine; Gly), AAA (for Lysine; Lys) (Supplementary Table S3). Our analyses showed that the codon usage patterns were highly similar among the 12 mitogenomes, with the most commonly used codon for each amino acid all being the same across all 12 strains (Figure 5). The bias in high A + T ratios in these mitogenomes have likely contributed to the preference of codons with high A + T content.

### Evolutionary Rates of PCGs and Nuclear Genes

The mean nucleotide frequencies of the three analyzed nuclear genes are A = 24.66%, T/U = 24.70%, C = 26.38%, and G = 24.27%, and those of mitogenomic PCGs are 29.31, 41.12, 14.03, and 15.54%, respectively. The rates of transitional and transversional substitutions among mitochondrial and nuclear genes vary, with the rates of transitional substitutions from T to C and from A to G at much higher frequencies in nuclear genes than in mitochondrial genes. Similarly, much higher rates of transversional substitutions from A to C, T to G, C to G and G to C were found in nuclear genes than mitochondrial genes. In contrast, rates for remaining types of transitional and transversional substitutions were found much higher in mitogenomes than in nuclear genomes of the same group of species (Table 2).

**TABLE 2:** Probability of substitution (*r*) from one base (row) to another base (column).

a. Mitochondrial genes				
From\To	A	T	C	G
A	–	*8.2627*	*2.8185*	**8.6270**
T	*5.8906*	–	**8.8802**	*3.1237*
C	*5.8906*	**26.0330**	–	*3.1237*
G	**16.2688**	*8.2627*	*2.8185*	–

**b. Nuclear genes**				
**From\To**	**A**	**T**	**C**	**G**

A	–	*3.8228*	*4.084*	**10.74**
T	*3.8172*	–	**24.48**	*3.756*
C	*3.8172*	**22.915**	–	*3.756*
G	**10.911**	*3.8228*	*4.084*	–

*For simplicity, the sum of *r*-values is made equal to 100. Rates of different transitional substitutions are shown in bold and those of transversional substitutions are shown in *italics*.*

**FIGURE 6:**
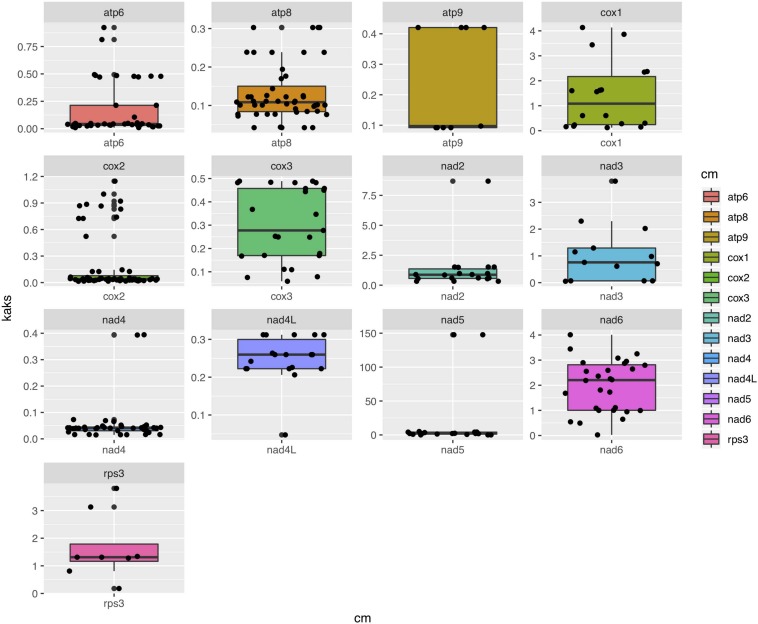
Box-plot comparisons of the Ka/Ks ratios estimated in the various mitochondrial genes. The estimates were based on pairwise alignments of samples.

Among the 15 mitochondrial PCGs, seven (*rps3, cox1-2, nad2-3*, and *nad5-6*) had a higher non-synonymous substitution rate (Ka) than synonymous substitution rate (Ks) between several species pairs. Our results indicate that besides purifying selection, NTF species were subject to positive selection at specific gene loci (Figure 6).

### Phylogenetic Analysis

**FIGURE 7:**
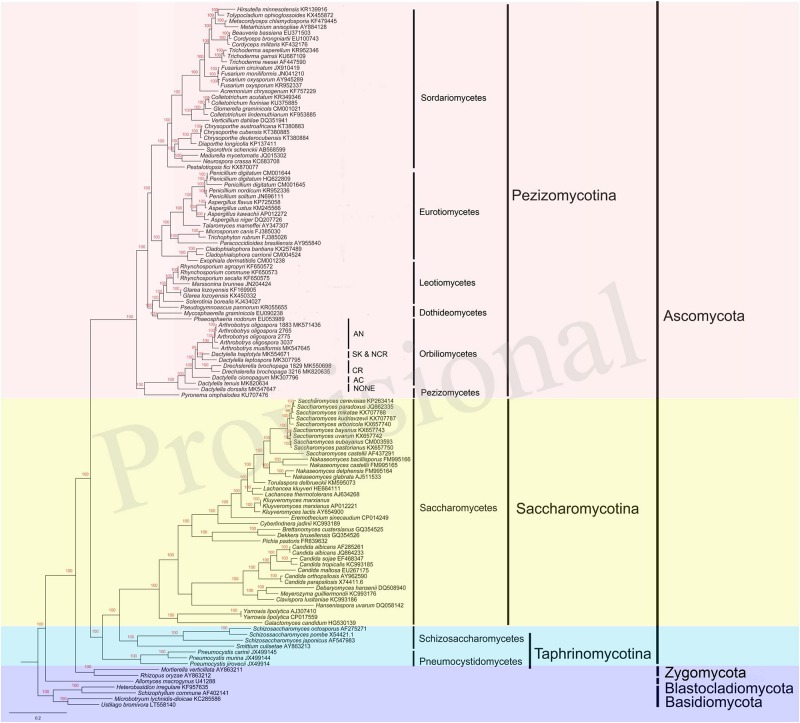
Bayesian tree of 104 species from the five main classes of Pezizomycotina, as well as representative species from other fungal phyla. The tree was constructed based on concatenated amino acid sequences of 14 conserved protein coding genes in fungi *atp6, 8–9, cob, cox1–3, nad1–6*, and *nad4L*. The resulting Bayesian posterior probabilities (BPP) ≥ 70% are shown above internal branches. GenBank accession numbers of all sequences are given. AN, adhesive networks; AK & NCR, Adhesive knobs and non-constricting rings; CR, constricting rings; AC, adhesive column; NONE, no trapping device produced.

Our single gene based phylogenetic analyses revealed no evidence for statistically significant incongruences among the 14 protein-coding gene trees. Thus, here we focus on analyzing the concatenated mitochondrial protein sequences. Our results revealed many well-support backbone nodes in Ascomycota, similar to those revealed by nuclear genes (Schoch et al., 2009; Figure 7). For example, high statistical supports were found for subclasses Saccharomycotina and Pezizomycotina. Interestingly, Saccharomycotina was found to be more closely related to Taphrinomycotina than to Pezizomycotina. Data presented here suggested that the common ancestor of Orbiliomycetes and Pezizomycetes likely represented an early diverging lineage of the Pezizomycotina (James et al., 2006), with the remaining four classes sampled here forming a well-supported clade. Moreover, in both the mitochondrial and nuclear phylogenies, these NTFs were found to be within a well-supported monophyletic group (Li et al., 2005; Yang et al., 2007). Our results suggest that non-predatory species with similar morphological characters (except the traps) to these NTFs (such as our DT and DD), may have differentiated from other Orbiliomycetes at an early stage during evolution. In our phylogeny based on concatenated mitochondrial protein sequences, the species producing adhesive column (DC) was found to be the first one diverging from the non-predatory species DT and DD. Therefore, DC likely represents one of the early branching members of NTFs. Other predators producing constricting rings and adhesive knobs and networks seemed to form a well-support clade that emerged later.

## Discussion

In this study, we obtained the mitogenome sequences of three strains of *A. oligospora.* These three mitogenome sequences were analyzed together with nine previously published mitochondrial genomes. Together, these 12 mitogenomes represent six species of NTF and two related species of non-predatory fungi. Our analyses indicated that these 12 fungal mitogenomes are among the largest and most variable in length of the known eukaryotic mitogenomes (Paquin et al., 1997; Salavirta et al., 2014). The size of published fungal mitogenomes varies from 12.06 kb to 235.85 kb, which represents a 19.6-fold difference in size. Likewise, great mitogenome size variations (146–280 kb) were revealed among NTF species, largely due to differences in the number and size of introns (28–55 introns per species).

Interestingly, the NTFs and their closely related two non-predatory fungal species are among the species with large mitogenomes, with strain DB29 having the largest mitogenome of all known fungi sequenced to date. Furthermore, two strains of the same species, DB29 and DB16, showed a large difference in their genome sizes, 280,699 and 193,195 bp, respectively. Based on mitogenome sequence comparisons, almost all the size differences among the 12 mitogenomes could be attributed to mitochondrial introns. However, it’s currently not known why certain strains and species accumulate more introns than other strains and species. Here, the two strains of *Dr. brochopaga* with different mitogenome sizes came from different sources: DB29 was isolated from the ascospore germination culture of its teleomorphic form *Orbilia orientalis* (Raitv.) Baral (Yu et al., 2006), whereas DB16 was directly obtained from a single conidium in a soil sample (Guo et al., 2009). In the lab, strain DB29 is capable of sexual reproduction while strain DB16 is incapable of sexual reproduction. It’s tempting to speculate that the ability of strain DB29 to reproduce sexually through mating may have contributed to the spread of introns to its mitogenome and consequently to its large mitogenome size.

Similarly, the mitogenome size differences among strains of *A. oligospora* were primarily due to the size and distribution of introns. In *A. oligospora*, strains belong to one of two mating types. The mating type genes regulate mating (hyphal fusion) and meiosis. Recent research has shown increasing evidence for mitochondrial DNA recombination under both laboratory and field conditions, though it is widely believed that the inheritance of mitochondrial DNA in fungi is uniparental and non-recombining (Wilson and Xu, 2012; Breton and Stewart, 2015; Xu and Wang, 2015; Wang et al., 2017). Indeed, mutation of a specific gene involved in sex-determination caused the rapid spread of multiple introns in the human pathogenic yeast *Cryptococcus neoformans* (Yan et al., 2018). In the case of NTF and their close relatives, further studies are needed to investigate the mobility of introns in genetic crosses. In addition, mitochondrial- and nuclear-associated plasmids and double-stranded RNA (dsRNA) elements are common in fungi and they can spread during hyphal fusion, potentially contributing to the diversity and size expansion in the mitogenomes observed in this study.

Because all mitogenomes are believed to have originated from a common ancestor, the mitochondrial gene order can also reflect the evolutionary relationships among organisms (Zheng et al., 2018). A comparative alignment of 12 mitogenomes with the Mauve program clearly showed a largely syntenic relationship among all samples we investigated, but the sizes and relative positions of homologous fragments showed slight variation among some of the species. Here, the two non-predatory species had a distinct location for *atp8* that’s different from that in other species. Closely related plants often contain mitochondrial genomes with different gene orders, which have been attributed to homologous recombination within individual mitochondrial genomes mediated by their high proportions of repeat sequences (Maréchal and Brisson, 2010). However, despite their potential importance, the mitochondrial gene order variations among fungi have rarely been evaluated (Aguileta et al., 2014). It is possible that the DD and DT gene arrangement represents the ancestral condition and those of the NTF are an evolutionarily derived condition. As indicated in the phylogeny, DD is located on the basal branch in the Orbiliomycetes clade, this change in gene order could be caused by an intra-molecular recombination within the mitochondrial genome of the common ancestor of the analyzed NTF.

The GC content of mitochondrial genomes varies among organisms, and can be affected by mutation bias, selection, and biases of reconstitution-related DNA repair (Chen et al., 2014). Interestingly, in almost all fungal mitogenomes sequenced so far, codon usage is biased strongly toward codons ending in A or T (Li et al., 2018a, b). Indeed, over 94% of the optimal codons in the 10 NTF mitogenomes end in A or T (Supplementary Table S2). This was likely due to the high AT content found in fungal mitogenomes, where the rates of transitional and transversional substitution from C, G to T, A are much higher than those of the reverse direction and those of nuclear genes.

As one of the most important organelles, mitochondrion generates the universal energy currency ATP and plays an important role in almost all biological activities. Even though mitochondria were originated from a common ancestral alpha- proteobacterium, they may have evolved together with different nuclear genomes and adapted to different environments (Yan et al., 2007; Einer et al., 2017; Zhang B. et al., 2017). Previous studies have shown that different groups of eukaryotes seem to have different mitogenome mutation rates, and the rates of mutation in fungal mitochondrial genomes are generally considered intermediate between those in animals (highest mutation rate) and plants (lowest mutation rate) (Aguileta et al., 2014). However, there are significant differences among fungal groups in their relative nuclear-mitochondrial genome mutation rates (Sandor et al., 2018). Regardless, mitochondrial genes have been used for evolutionary studies in a variety of fungi, such as the endophytic fungus *Phialocephala scopiformis* (Robicheau et al., 2016), the entomopathogens *Hirsutella vermicola* (Zhang Y.-J. et al., 2017), *Pochonia chlamydosporia* (Lin et al., 2015), and *H. thompsonii* (Wang et al., 2018). For example, a mitogenomic phylogeny showed that all of the invertebrate-pathogenic fungi cluster together to form a monophyletic group in Hypocreales, which is noticeably distinguished from a cluster comprising of plant fungal pathogens (Lin et al., 2015). Based on mitochondrial gene sequences, our NTFs were also found to be in a well-supported monophyletic group different from the two non-predatory species, consistent with these invertebrate-pathogenic fungi sharing a common ancestor.

At present, there are several hypotheses on the origin and evolution of nematode-trapping lifestyles in the Ascomycota, and these hypotheses differ in the order of emergence of individual trap types, including what the ancestral trap type might be (Rubner, 1996; Ahren et al., 1998; Li et al., 2005; Yang et al., 2007). Based on the phylogenetic relationship revealed in our study, predatory fungi evolved from non-predatory ancestors and we propose that adhesive column was likely the first type to emerge. This was then followed by the emergence of constricting rings (CR). Our results are consistent with the hypothesis of Yang et al. (2007) that the trapping mechanisms within the Orbiliales have evolved along two major lineages, one leading to species with constricting rings and the other to species with adhesive traps, including three-dimensional networks, knobs, and branches. Indeed, physiological and microscopic observations indicate that CR and various adhesive devices have distinctly different materials. For example, microscopically within trap cells, CR contains unique, oblong-shaped, electron-dense inclusions, which are absent in the ring cells after nematodes are captured, whereas cells of adhesive devices exhibit multiple globose electron-dense bodies that persist (Tzean and Estey, 1981; Nordbring-Hertz, 1988). Our analyses demonstrated that, though with similar morphology, non-constricting rings (NCR) were phylogenetically distant from the CR, which is consistent with the phylogeny reconstructed based on multiple nuclear gene sequences. Although the rates of transitional and transversional substitutions between mitochondrial and nuclear genes varied among our study organisms, the framework established by the mitogenome sequences showed that the origins and divergence patterns of various predatory fungi in Ascomycota were similar to those inferred based on nuclear gene sequences. Overall, our results indicate that mitochondrial genes can be useful molecular markers for NTF taxonomy, systematics, population genetics, and evolutionary studies.

Due to their functional constraints, mitochondrially encoded protein sequences typically evolve slower than predicted by the high nucleotide substitution rate in the mitochondrial genome (Giorgi et al., 1999). Thus, we expected to observe prevalent evidence for purifying selection on non-synonymous mutations during mitochondrial evolution (Meiklejohn et al., 2007). However, our analyses showed a mixed pattern. Interestingly, a recent analysis of diverse animal taxa showed that 26% of non-synonymous mitochondrial substitutions may have been fixed by adaptive evolution (James et al., 2015). Furthermore, frequent non-synonymous polymorphisms in both *atp6* and *VAR1* genes were observed in yeast, consistent with relaxed purifying selection on either protein-coding genes or the intergenic regions (Jung et al., 2012), resulting in fast evolution of yeast mitochondrial genomes. Similar to most yeasts, NTF can grow as saprophytes in soils. However, different from yeasts, NTF can enter the parasitic stage by developing specific traps. During the switch between different lifestyles, multiple fungal signal transduction pathways are activated by its nematode prey to further regulate downstream genes associated with diverse cellular processes such as energy metabolism, biosynthesis of the cell wall and adhesive proteins, cell division, glycerol accumulation, and peroxisome biogenesis (Yang et al., 2011; Meerupati et al., 2013). Therefore, genes involved in energy metabolism (e.g., cytochrome c oxidase, ATP synthase and NADH dehydrogenase subunits) might be targets of natural selection and adaptation to meet the huge change in energy demand, facilitating genetic changes in these genes.

Other than these energy metabolism-related genes with high Ka/Ks ratios, our analyses also showed a high Ka/Ks ratio in the *rps3* gene among the 12 mitogenomes. Interestingly, the mushroom species *Hypsizygus marmoreus* also has a high Ka/Ks ratio (1.4) in *rps*3 gene, consistent with positive selection of this gene and its functional importance (Wang G. et al., 2019). Similarly, the *rps3* gene in the entomopathogenic fungus *P. chlamydosporia* has also likely experienced positive selection, leading to a unique evolutionary pattern in Hypocreales (Lin et al., 2015). The RPS3 protein plays a critical role in ribosome biogenesis and DNA repair in eukaryotes (Kim et al., 2013). This is the only mitochondrial ribosomal protein encoded by mitogenomes in NTFs. It’s currently unknown why this protein underwent diversifying selection among several distinct groups of fungi. This and other issues such as the relevance of our findings on NTF mitogenomes to other functionally important groups of fungi (e.g., those parasitizing insects, plants, and other animals) remain to be investigated.

## Data Availability Statement

The datasets generated in this study can be found in GenBank (Accession number MK571436).

## Author Contributions

JX, ZY, K-QZ, and YZ conceived and designed the study. YZ and JX wrote the manuscript. GY, CD, and MF conducted the experiments. YZ, GY, CD and MF analyzed the data. YZ and JX revised the manuscript. All authors read and approved the final manuscript.

## Conflict of Interest

The authors declare that the research was conducted in the absence of any commercial or financial relationships that could be construed as a potential conflict of interest.

## Footnotes

1 https://www.ncbi.nlm.nih.gov/genomes/GenomesGroup.cgi?opt=organelle&taxid4751&sort=Genome

2 https://megasun.bch.umontreal.ca/cgi-bin/dev_mfa/mfannotInterface.pl

3 https://www.ncbi.nlm.nih.gov/genomes/MICROBES/glimmer_3.cgi

4 https://www.ncbi.nlm.nih.gov/orffinder/

5 http://codonw.sourceforge.net/index.html

## Supplementary Material

TABLE S1 Dataset of AT skew, GC skew, GC content and length of the 15 protein coding genes in the 12 analyzed mitogenomes.

TABLE S2 Locations of the rps3 gene in each of the 12 analyzed mitogenomes.

TABLE S3 Dataset of the relative synonymous codon usage (RSCU) in the 14 mitochondrial protein-coding genes.
